# Exploring the motivations for rural tourism in China during the COVID-19: The existence of a single motivation

**DOI:** 10.1371/journal.pone.0294610

**Published:** 2023-12-06

**Authors:** Songting Zhang, Yichao Wu, Wen Bao

**Affiliations:** 1 School of History, Culture and Tourism, Fuyang Normal University, Fuyang, Anhui Province, China; 2 Yuexiu Institute of Hospitality Administration, Zhejiang Yuexiu University, Shaoxing, Zhejiang Province, China; 3 Department of Tourism, Lanzhou Vocational and Technical College, Lanzhou, Gansu Province, China; Fiji National University, FIJI

## Abstract

The COVID-19 epidemic had an appropriate impact on tourists’ trip psychology and their subsequent behavior in participating in rural tourism activities. The purpose of this paper is to explore the types of motivations Chinese tourists have for participating in rural tourism in the context of COVID-19, and to comparatively analyze the similarities and differences in motivations for rural tourism during the epidemic and in normal times. An interpretive paradigm qualitative data collection method was used: semi-structured interviews and focus group discussions. Respondents were 21 tourists, who were selected through purposive and snowball sampling. Through content analysis, we found that rural tourism motivations during the epidemic included both diversified and singular motivations. The pull effect of rural destinations is related to distance and ease of realization. For rural areas in close proximity, a single motivation is sufficient to drive tourists. In addition, we found that there was no "altruistic motivation" for rural tourism during the COVID-19 period, but "altruistic feelings" for the preservation of ancient villages were generated during rural tourism. Finally, we discuss the theoretical and practical significance of this study and make suggestions for future research. The study explains tourists’ companionship preferences, activity choices, and affective changes, and provides a basis for the operation and advertising strategies of rural destinations to attract tourists and promote their sustainable development.

## Introduction

In early 2020, the COVID 19 outbreak and the global pandemic had a significant impact on tourism, leading to significant changes in mobility, social behavior, consumption patterns and leisure [1]. With improved pandemic prevention and control capabilities and widespread vaccination, China’s tourism industry, although it has come out of the bottom of the valley and urban micro-vacation and rural tourism are growing rapidly, still faces major uncertainties and challenges. It is therefore crucial to understand how pandemics affect tourist behavior and decision-making processes [2, 3] in order to lead the recovery of Chinese tourism. This study explores an initial step to understand the psychology and motivation of tourists’ trips during the COVID-19 years.

**Fig 1:**
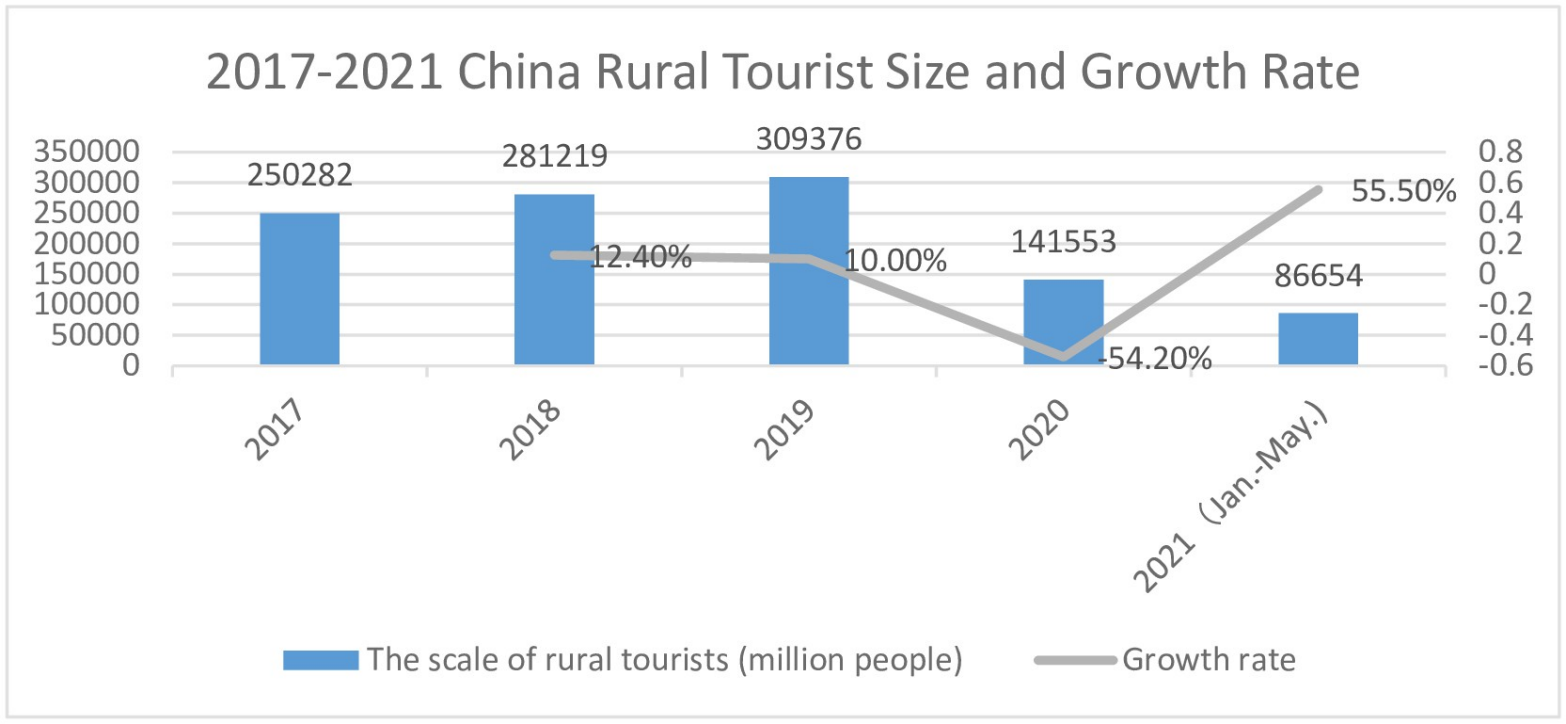
2017–2021 China rural tourist size and growth rate. (A) The blue bar represents the scale of rural tourists. (B) The gray line represents the growth rate. *Source: Adapted from China Tourism Research Institute, Smart Research Consulting (2021-11-23)*, https://www.chyxx.com/industry/202111/986974.html.

National restrictions triggered by the COVID-19 pandemic affect tourists’ outdoor recreation behaviors and may have a profound psychological impact on tourists’ thinking, feelings, and emotions, thus altering the tourists’ tourism decision-making process. Tourists’ travel decisions can be influenced by subjective evaluations and destination-related information [4]. In addition, work styles with more free time, such as online work, provide supportive conditions for people to travel. After the epidemic was effectively controlled, rural tourism around cities recovered rapidly by virtue of the advantages of low density, proximity to nature, and short distances [5] (Fig 1). Since 2022, rural tourism has recovered 92% compared to the same period in 2019, making it one of the tourism categories with the strongest recovery momentum. Anhui Province, located in central China, is a traditional agricultural province with rich agricultural and rural resources [6]. The region is close to the economically developed and densely populated Yangtze River Delta region, with a large consumption capacity for rural tourism, and during the epidemic, rural tourism in Anhui Province showed explosive growth.

During the epidemic period, using Anhui Province as a case study, exploring the motivation of rural tourism is conducive to discovering people’s special needs, companion preferences, and psychological changes in the special period, and to further promote the development of rural tourism and then lead to the overall recovery of tourism. This study adopts the qualitative research method of semi-structured interviews and focus groups, aiming to comprehensively discover and deeply understand the motivation of rural tourism. The structure of this paper is as follows: first, a brief review of rural tourism, motivation theories and research on the impact of disasters on tourists’ motivation was presented, and the research questions of this study were put forward. Next, the choice of methodology and the data collection process were explained. Then, the results of data analysis were shown in the findings section. Finally, the results were discussed in detail and the implications and limitations of the study are pointed out.

## Literature review

### Rural tourism and development

The term "rural tourism" in English literature mainly includes "rural tourism", "farm tourism" and "village tourism" [7]. Currently, the definition of rural tourism remains inconsistent between developed and developing countries. In general, the connotation of rural tourism generally includes four key components: location, sustainable development, community characteristics, and experience [8].

The benefits of rural tourism as an engine of economic development and a contributor to the quality of life of rural residents are often emphasized, and it is one of the fastest growing types of tourism in recent decades [9, 10]. Rural tourism can meet the psychological needs of people to enjoy the natural scenery, has the advantages of low tourism cost, short time consumption, low traffic density, and high safety, and is valued by society as an important industry to promote rural economic development [11]. Based on the perspective of rural tourists, it was found that due to the restorative and therapeutic power of rural nature, it can improve physical and mental health, and can satisfy the needs of people escaping from urbanization and industrialization, looking for natural scenery, and enjoying the countryside customs and folklore [12, 13].

### Motivation theory and rural tourism demand

The earliest research on motivation can be traced back to 1932, when the British scholar Tolman, Hall, and Bretnall (1932) proposed that motivation includes intrinsic motivation driven by underlying emotions and extrinsic motivation attracted by cognitive level [14]. The study of tourism motivation began in the 1950s, and tourism motivation is the intrinsic motivation that drives tourists to produce tourism behavior, which is one of the important elements of consumer behavior research and tourism research since decades [15]. Currently, the more influential theories of travel motivation include Maslow’s 5-stage theory on motivation [16], Dann’s “Push-Pull” motivation Theory [17], Crompton’s Vacation Motivation [18], Iso-Ahola’s model of the social psychology of tourism [19], and Sharpley’s “Internal-External” Motivation Drive Theory [20].

Dann (1977) proposed a "push-pull" theory of tourism motivation. He argued that tourists’ motivation to travel consists of push and pull, and any intrinsic factor that can motivate travel can be regarded as push motivation, while those factors that can attract people to travel, such as destination attributes, can be regarded as pull motivation [21]. This theory is the classic theory of motivation, easy to understand, and has been verified and supported by domestic and international empirical studies over the years. Pesonen, Komppula, Kronenberg, and Peters (2011) applied “push-pull” motivation theory to two different rural destinations and found significant differences in push-pull motivation between the two regions by comparing them, with visitors driven by different variables apparently searching for different destination attributes [22].

Rural tourism motivation is a complex psychological structure of tourists in the whole process of rural tourism, and its research is mainly conducted based on theories related to tourism motivation in combination with the type of rural tourism and market segmentation of tourists [23]. Park and Yoon (2009) concluded that Korean rural tourists can be categorized into four groups: family reunion seekers, passive tourists, want-everything seekers, and learning and excitement seekers [24]. Rid, Ezeuduji, and Pröbstl-Haider (2014) categorized rural tourists in The Gambia into: heritage and nature seekers, multiple experience seekers, multi-experience and beach seekers and sun and beach seekers [25].

Tourists’ desire to escape the stress of the city is the main motivation for them to choose to travel to the countryside [11, 24, 26]. Visiting relatives and friends is also one of the social motivations, which is related to the rapid development of urbanization: a large number of rural people have moved to cities, but their blood ties and geographical ties with the countryside have not been broken, and tourism has become a bond to maintain interpersonal relations [27]. There are usually multiple motivations to promote rural tourism [24].

### The impact of crisis events on tourism demand

Crisis events such as terrorist attacks, crime and financial crises all have an impact on tourism. As long as terrorist attacks occur, tourism demand will continue to decrease, and tourism will eventually come to a standstill, no matter how severe the terrorist activity is [28]. If tourists fail to anticipate financial crises or are insensitive to them, no precautionary measures will be taken and tourism demand will not decline, as was the case in 2001 when the tourism industry in Northern Cyprus was not affected by the financial crisis [29]. In the context of the financial crisis, the development of activities such as festival tourism and business trips would have contributed to the growth of immediate tourist demand and the realization of an increase in the number of tourists. It has been noted that the impact of crisis events on the demand for tourism is not as great as expected. When choosing a destination, tourists usually avoid terrorist locations and seek places where the political situation is stable to ensure security [30].

Affected by natural disasters such as hurricanes, earthquakes, volcanic eruptions, tsunamis, etc., tourism is limited [31–33]. For destinations devastated by natural disasters, the most striking feature is generally a decrease in tourist arrivals [34–36]. Tourists generally prefer to travel with their close family or friends after a natural disaster [37], and their perception of risk varies according to socio-demographic characteristics such as age, gender, and education [38]. In addition, tourists’ familiarity with the destination also influences their perception of risk, which in turn affects their travel needs and decisions.

Studies analyzing the impact of the 2003 SARS outbreak in China on tourist behavior have found considerable similarities to the current pandemic because of the nature of the virus and the nature of the restrictions imposed. During an outbreak, most people are likely to travel with people they know, including family and relatives, and companionship preferences will change somewhat [39]. Activities with less human contact and demand for nature and ecotourism will be more popular. Tourists are more likely to consider health and safety when traveling, and there are differences in the perceived impact of SARS on travel intentions, behaviors, and patterns among different demographic characteristics, with healthcare workers and relatives being more conservative about the virus. Others such as Ebola, avian flu and pandemic influenza show similar effects [40]. After the occurrence of COVID-19, which brought local or regional restrictions, there were some changes in the motivation of rural tourists, who considered rural tourism to be outdoors, safer, and the rural environment combining nature, cuisine, and culture as a good place to visit during the epidemic [41].

### Research gaps

Early studies of COVID-19 focused on investigating the effects of risk perception on behavior and responses to tourism restrictions, social costs, and tourism recovery strategies. In studies of tourists’ decision-making behavior, empirical studies of variable relationships were conducted using traditional variables such as attitudes and motivations in combination with risk perceptions and participation intentions brought about by COVID-19. These studies rarely provide a grounded view of what is considered from the tourists’ perspective, and cannot fully portray a complete picture of the strategies developed under the influence of social systems and individual factors.

In order to get the reasons for the popularity of rural tourism during COVID-19 and to explore a complete and comprehensive picture of tourists’ individual needs and motivations, this study captures first-hand information from tourists through interviews and focus group discussions to obtain the reasons and motivations for tourists’ choice of rural tourism during COVID-19. In other words, this study aims to find answers to the following research questions: -What motivates tourists to participate in rural travel during the current COVID-19 pandemic? -How do tourists differ from the normative context in terms of rural travel motivation in the extraordinary context of COVID-19?

## Methodology

Since the main aim of this study was to gain a rich and in-depth understanding of the motivations of rural tourists, rather than to determine causal relationships between variables, the interpretive paradigm of qualitative research was used. Scholars believe that qualitative research paradigm can explain behavioral process more fully [42, 43], compared with quantitative research methods, qualitative research paradigm is more able to describe and explain in detail the types of rural tourism motivation. Prior to the main study, researchers conducted a half-month pilot study to test the validity of the interview guides and to ensure that they adequately collected the data needed to answer the research questions. The main study was conducted over a three-month period from July to October 2022.

### Data collection

Respondents were drawn from both purposive and snowball sampling, and 21 respondents participated in the study: 15 were tourists nearing the end of their rural tourism activities, and the other six were rural tourism enthusiasts (having participated in at least one rural tourism activity per year for the last three years). The main study included two methods of data collection: semi-structured interviews and focus groups. During the study period, the outbreaks were uninterruptedly present in different locations, so the collection methods included both online and offline methods.

Data saturation should be an explicit goal when determining the sample size [44]. The judgment and experience of the researcher in evaluating the quality of the information collected determines the sample size [45]. Therefore, during the data collection process, the number of informants reaches the research needs when new categories or topics no longer emerge from the data, at which point the data is saturated [46]. Failure to achieve data saturation affects the quality of ongoing research and hinders the validity of content [47]. Therefore, in this study, the researcher continued to collect data until no new information was available, that is, until data saturation was reached. As a result, it was ultimately determined that data saturation had been reached for 21 respondents.

For respondents, see Table 1 Full demographic details of respondents and information about interviews, 1–6 participated in the pilot study and all 1–21 participated in the main study.

**Table 1:** Full demographic details of respondents and information about interviews.

Name	Age	Gender	Marital Status	Education Level	Occupation	Interview Date	Interview Duration
T01	45	M	Divorced	Bachelor degree	State enterprise employee	20/07/2022	57mins
T02	44	M	Married	Vocational college graduate	Tourism practitioner	23/07/2022	55mins
T03	32	F	Married	Bachelor degree	Teacher	25/07/2022	63mins
T04	22	M	Single	Bachelor degree/studying	College students’	27/07/2022	53mins
T05	23	M	Single	Bachelor degree/studying	College students’	27/07/2022	69mins
T06	37	M	Married	master’s degree	Civil Servicer	10/08/2022	70mins
T07	19	F	Single	Bachelor degree/studying	College students’	10/08/2022	49mins
T08	23	F	Single	Bachelor’s degree	Teacher	15/08/2022	40mins
T09	22	F	Single	Bachelor degree/studying	College students’	10/08/2022	48mins
T10	51	F	Married	Junior Mid- School	Retired employees	17/08/2022	50mins
T11	53	M	Married	master’s degree	University teacher	20/08/2022	44mins
T12	25	M	Single	PhD in progress	PhD student	28/08/2022	55mins
T13	45	M	Married	master’s degree	Civil Servicer	28/08/2022	37mins
T14	39	F	Divorced	Bachelor’s degree	Police officer	30/08/2022	67mins
T15	30	M	Married	master’s degree	Teacher	04/09/2022	53mins
T16	40	F	Married	PhD in progress	University teacher	13/09/2022	66mins
T17	44	F	Widowed	master’s degree	Teacher	15/09/2022	58mins
T18	37	F	Married	Junior Mid- School	Businesswoman	16/09/2022	49mins
T19	55	F	Married	Tertiary education	Civil Service	30/09/2022	51mins
T20	50	F	Married	Tertiary education	Civil Service	01/10/2022	53mins
T21	22	F	Single	Master’s degree in progress	Graduate Student (Tourism practitioner)	10/10/2022	61mins

Note

Gender: M = Male; F = Female

All interviewees were interviewed independently

Nos.3,6,19,20,21 interviews were conducted online via Tencent Conference.

Nos.1-6 are research respondents from the pilot study.

Nos.7-21 are research respondents from the main study.

Nos.4,5,12,16 respondents were participants in the focus group interviews, which took place on October 16, 2022 on the online Tencent meeting platform and lasted one hour and 35 minutes. (The researcher had been studying in Korea since September 20, 2022)

Interviews and focus group discussions were audio-recorded with the consent of the interviewees. Since both the researcher and the respondents were Chinese, the researcher was able to accurately determine what the respondents meant based on the situation; even if the respondents partially answered the questions in dialect or slang. After the transcription was completed, the researcher emailed the text to the respondent for confirmation to determine accuracy.

### Data analysis

This study utilized the grounded theory to qualitatively analyze the data through three steps of open coding, spindle coding and selective coding, and the theoretical saturation test to ensure the validity of the study. According to the proportion of one-third, this study did two independent coding of the interviews separately, without affecting each other. First, the interview transcripts were studied and analyzed individually without affecting each other, and sentences closely related to the study content were selected for conceptualization, and the concepts were classified and further categorized. Finally, the respective codes were compared one by one, identifying two independent codes, codes with the same and different code content, using the same code content, and reading and comparing the different codes for in-depth reflection. Comparisons are made on the basis of a large number of papers and in this process, concepts that occur less than twice are excluded from categorization.

#### Open coding to extract concepts and categories

Open coding is the process of breaking down, comparing, conceptualizing, and categorizing data collected at the beginning of a study, that is, by breaking up large amounts of data, assigning concepts to them, and then reassembling them in new ways so that they can be manipulated according to certain principles [48]. Its purpose is to discover the same or similar types from the collected primary data, and at the same time name the types in order to determine the concepts and dimensions of the types. Open coding consists of 3 steps: (1) conceptualization, extracting the contents from the original comments, breaking them into independent sentences, and extracting coding elements from these sentences, which in turn leads to the transformation of generalized language to refined language and the formation of preliminary concepts; (2) concept categorization, optimizing, analyzing, and filtering the concepts, aggregating concepts in the same category of genera, and analyzing the connections between words to form concept clusters belonging to the same category; (3) categorization, further abstracting and naming the concept clusters. We utilized Nvivo 14.0 to conduct verbatim reading, coding and labeling the collected interview data verbatim without any researcher’s preconceptions and biases, to generate initial concepts and discover conceptual categories from the raw data. The results of open coding are shown in Table 2 The results of open coding. In this paper, open coding was utilized to obtain 20 initial categories with a total of 112 nodes, of which the top ten with high frequency were Leisure and relaxation, friends outdoor reunion, family fun and parent-child education, implicitly of the aboriginal people, purchase of agricultural products, rural specialty Lodging, agricultural activities experience, rural recreational environments and outdoor spaces, specialty agricultural product, as a hot spot.

**Table 2:** The results of open coding.

Interview content	Scope of open code extraction	Frequency
*Every year in the spring and autumn*, *I take a walk in the countryside to relax and appreciate life on the long walks*.	Leisure and relaxation	22
*To satisfy their "nostalgia complex"*, *experience the countryside life*, *feel the countryside culture*, *but also want to eat the authentic countryside food*, *see the new look of the new socialist countryside today*.	Nostalgia	3
*Thirdly*, *I hope to be able to reflect and remember in the process of rural tourism and relaxation*, *to discover my own problems or to reflect on myself*.	Self-reflection	1
*My main motivation for choosing rural tourism is health care*, *retirement and relaxation*, *and the ability to take a quiet vacation*.	Health and retirement	1
*The main motivation for this trip was nature sightseeing*, *supplemented by sports and a taste of the countryside cuisine*.	Outdoor activities and fitness	2
*The third is about colleagues*, *classmates to an exchange trip*, *or a friendship trip*	Friends outdoor reuniol	4
*I mainly accompanied my children to the countryside on weekends*. *Sometimes*, *in order to complete the practical homework assigned by the teacher*, *I helped my children experience the rural life*	Family fun and parent-child education	6
*The people there are simple*, *the way of life is very primitive*, *and the villagers are happy and joyful*.	implicity of the aboriginal people	6
*In addition*, *it is relative to tourism to have some understanding*, *but also the purpose of visiting the study tour*	Inspection of cooperative projects	2
*I also went to a vineyard in the afternoon*, *grape varieties*, *planting area is also large*. *Farmers will bring out their own wine for us to taste and sell*	Purchase of agricultural products	5
*I mainly accompanied my children to the countryside on weekends*. *Sometimes*, *in order to complete the practical homework assigned by the teacher*, *I helped my children experience the rural life*.	Complete practical assignments	1
*I feel the scenery is good through wechat moments and Douyin recommendations*, *and I want to clock in with my family*. *Take a look at nature*, *relax and do some parent-child activities*.	Focus on Fashion	1
*In addition to these*, *I will also consider villages with certain historical or strong cultural characteristics*, *such as the former residences of some celebrities or some old revolutionary areas*.	Rural Specialty Lodging	8
*I often consciously go to the countryside with my classmates and friends*, *and eat farmhouse*	Agricultural Activities Experience	16
*The third is that we hope the children can be educated while playing*, *such as farming culture*, *folk customs and red culture*.	Specialty Cultural Accommodations	4
*I like natural scenery more than artificial scenery*, *I often travel*, *focusing on the choice of fresh air*, *beautiful environment of the countryside*, *which prefer to have a humanistic heritage of ancient towns*, *most like the ancient town of Tengchong Heshun in Yunnan and Xidi*, *Hongcun in southern Anhui*, *Yunnan Miao Village*, *Hainan East undeveloped coastal villages*	Rural recreational environments and outdoor spaces	12
*The people there are simple*, *the way of life is very primitive*, *and the villagers are happy and joyful*.	Kind villagers	2
*In recent years*, *new agriculture and breeding industries have been developed*	Good projects to attract investment	4
*I was impressed by this trip because we rode our bicycles in the countryside*, *looking for nature*, *tasting the countryside food*, *picking grapes and catching fish by the river yesterday*	Specialty Agricultural Product	7
*My hometown*, *Anhui*, *there are many ancient villages*, *the kind of Hui-style architecture*, *green bricks and white tiles*, *looking from afar with the ink painting*, *itself is a very beautiful scenery*	As a hot spot	5

#### Axial coding

Open coding of text, line by line and sentence by sentence, is a process of identifying and developing concepts and their characteristics and dimensions. These steps include naming and categorizing similar events and situations, leading to the formation of categories, and ultimately to coding codes and lists of categories in Table 3 Axial coding codes and categories. In the open coding process of this study, a total of 20 original discourses and concepts were produced, on the basis of which 2 main categories were obtained through axial coding, namely "push" and "pull".

**Table 3:** Axial coding codes and categories.

Main category	Sub-category	Connotation of categories	Counterpart categories
**Push**	Psychological motivations Physical motivations Social-related motivations Other motivations	Tourists’ own driving factors influencing rural tourism motivation include psychological motivation Tourists’ own driving factors influencing rural tourism motivation include physical motivation Tourists’ own drivers influencing rural tourism motivations include socially relevant motivations. Tourists’ own driving factors influencing rural tourism motivation include other motivations.	Leisure and relaxation Nostalgia Self-reflection Health and retirement Outdoor activities and fitness Friends outdoor reuniol Family fun and parent-child education implicity of the aboriginal people Inspection of cooperative projects Purchase of agricultural products Complete practical assignments Focus on Fashion
**Pull**	Rural Specialty Lodging Agricultural Activities Experience Specialty Cultural Accommodations Rural recreational environments and outdoor spaces Kind villagers Other characteristic factors	Attractiveness of the rural tourist destination influences rural tourism motivation, including rural specialty accommodation Attractiveness of rural tourist places influences rural tourism motivation including agricultural activity experience Attractiveness of rural tourist places influences rural tourism motivation, including characteristic cultural accommodation Attractiveness of rural destinations influences rural tourism motivations, including rural recreational environments and outdoor spaces Attractiveness of a rural destination influences rural tourism motivation, including friendly villagers Attractiveness of rural tourism places influences rural tourism motivation, including other characteristic factors	Rural Specialty Lodging Agricultural Activities Experience Specialty Cultural Accommodations Rural recreational environments and outdoor spaces Kind villagers Good projects to attract investment Specialty Agricultural Product As a hot spot

#### Selective coding

Selective coding continues with axial coding at a higher level of abstraction, the purpose of which is to identify the core categories around which other proposed categories can be merged and integrated to form a complete "story line". In this paper, we have used selective coding to obtain one core main category, namely rural tourists motivations (Table 4).

**Table 4:** Selective coding and main category.

Main category	Sub-category	Frequency
**Rural tourists motivations**	Push Pull	54 58

## Discussion

After the COVID 19 pandemic was brought under some control, travel agencies, airlines and other travel intermediaries were out of business for a longer period of time, causing people to turn their attention to rural tourism in order to meet their outdoor needs, thus providing a valuable option for the rapid recovery of the rural tourism economy [49]. This study obtained the current motivations of rural tourists in China through semi-structured interviews and focus group discussions. These findings provide a basis for how COVID-19 influences tourists’ motivations, companionship preferences, and travel decisions.

### Outdoor countryside meets people’s travel needs during COVID-19

In the study, it was found that the negative effects of crowding were amplified during the spread of pathogens, and people were more likely to choose the outdoor countryside for their tourism activities. Moreover, to reduce psychological discomfort, people wanted to replace other overnight trips with day trips and trips to nearby countryside. This finding complements the theory of substitution proposed by Iso-Ahola (1986) [50], as well as the study by Hall and Shelby (2000) [51]. In other words, neighboring rural destinations were perceived as safe and convenient places to go during the epidemic. Currently, the motives of rural tourism mainly involve psychological motives, physical motives, social motives, characteristic experience motives and other single motives such as investigating cooperative projects and helping children to finish their homework. The psychological motivation of leisure and relaxation was mentioned several times among the psychological motivations of rural travelers, and most respondents expressed the desire to escape from the city and get away from stress, and they usually traveled with family and friends, and these findings support the view that rural tourism is preferred for relaxation and enhancing parent-child relationship based on safety and cost in the previous study.

In this study, respondents frequently used terms like "escape" and "evasion" to describe their motivation for rural travel, indicating a desire to detach from the distractions of their everyday urban environments. This is because in recent years, the high cost of education and housing prices in China’s cities and the fierce competition in the workplace have made city dwellers live in a stressful atmosphere, and they choose to travel to the countryside, which is different from the city, to enjoy the tranquility and comfort of rural life and to obtain both physical and psychological relaxation, which is another way of labor reproduction.

During the interviews, many interviewees mentioned that being in a rural environment can "calm oneself", "purify the mind", and "self-reflect", which shows that in a heterogeneous environment such as a village, which is very different from an urban environment It can be seen that in the heterogeneous environment of the countryside, which is very different from the urban environment, people’s bodies are relaxed and at the same time their minds are changed, and they try to think about their lives and find their true selves. Travel needs belong to the higher level of Maslow’s five needs theory, and the satisfaction of the spiritual aspect of the journey is also a need above the physiological needs and security needs. The resulting spiritual motivation has driven the demand for improved self-cognition and deeper travel experiences for domestic travelers in recent years, which in turn has influenced changes on the supply side and promoted high-quality enhancement of rural tourism products.

This study found that few Chinese rural tourists cited volunteer tourism motivations such as contributing to the rural community, but more cited health, fitness, and retirement motivations. In the study of rural tourist motivations. Chinese rural tourists described their motivations based on personal needs, with few tourists citing the motivation of "contributing to the rural community". However, rural tourists in Africa, which is also a developing region, are motivated by the desire to contribute to the community while enjoying the natural environment of the countryside [52]. The researchers argue that this does not mean that the Chinese do not have an "altruistic mentality" because the Chinese perceive volunteer tourism destinations as those that are poor, backward, and in need of help in some way. With the recent development, the village appearance, farmers’ income and living standard of Anhui villages have been improved dramatically. The Anhui countryside in the advertisement is quiet and idyllic, and pleasant farm life, which are hardly tourist motives to contribute to the community and help the countryside, but are more likely to inspire tourists to desire for idyllic leisure life.

In comparison with foreign studies of rural tourism motivations, it was found that the health, fitness, and retirement motivations were mentioned several times in Chinese rural tourism motivations. It can be seen that people who have lived in cities for a long time see the rural environment as a natural and pollution-free natural environment. As China’s aging process accelerates, people with money and leisure begin to pursue a healthier lifestyle. They believe that the countryside is free of industrial pollution, with clean air and good water quality, and that organic food such as agricultural and sideline products are good for health. In many places, "reverse urbanization" has occurred, and many urbanites have their "second homes" in the countryside.

### Single motivation can also drive rural tourism

In this study, a single rural tourism motivation was found to exist and be sufficient to drive rural tourism behavior. However, many scholars argue that rural tourism motivation is multidimensional and different tourists will have a structure of rural tourism motivation that does not pass [52–55]. Travel motivation is the driving force prior to the start of tourism activities, and the single motivations found in this study include visiting cooperative projects, helping children with practical assignments, buying scarce and special agricultural products, and chasing fashion—hitting the Netflix spots. The researcher believes there are two main reasons for this. First, the rural tourism place is distinctive and specialized in a certain area, for example: a village famous for mulberry fungus cultivation, a village with patriotic education features, and a specialty crop radish origin. On the other hand, the accessibility, proximity and convenience of rural tourism places make it possible for a single motive to be realized relatively quickly. This finding provides insights for rural tourism development: villages with large volumes and superior resource endowments can develop multiple rural tourism products to attract more tourists and satisfy their multidimensional tourism experiences; while villages with smaller volumes but distinctive characteristics can develop one rural tourism product to attract a targeted rural tourism market segment according to local conditions and focus.

In addition, during the study period, respondents were experiencing China’s strict "dynamic zero" epidemic prevention and control policy. During the containment period, people experienced anxiety during the containment period, compensatory travel during the post-closure period, and a stable period during normal times. In particular, during the post-closure compensatory period, respondents expressed the idea that "they had been holding it in for too long and just wanted to get some air", but due to China’s "no travel unless necessary" policy, respondents could only travel to the countryside around the city as a destination, which on the one hand However, due to China’s "no distant travel unless necessary" policy, respondents could only travel to rural areas around cities as destinations, which on the one hand meet the need for outdoor activities and relaxation, and on the other hand meet the requirements of epidemic prevention and control, avoiding the risk of long-distance infection. Therefore, the COVID-19 itself is not a motivation for tourism, but after the epidemic is sealed and controlled, it will give rise to some ideas such as "going out for a breath of fresh air" and "too depressing to escape", and these tourists want to get outdoors, and the countryside is a good place to do so. It can be found that due to the regional limitations of the epidemic and people’s concern about the spread of the virus, a safe, rural environment that combines nature, food and culture can satisfy and easily fulfill people’s tourism needs during the epidemic, which will also be a good opportunity for the development of global rural tourism.

### Rural tourism motivation in the framework of the "push and pull" theory

**Fig 2:**
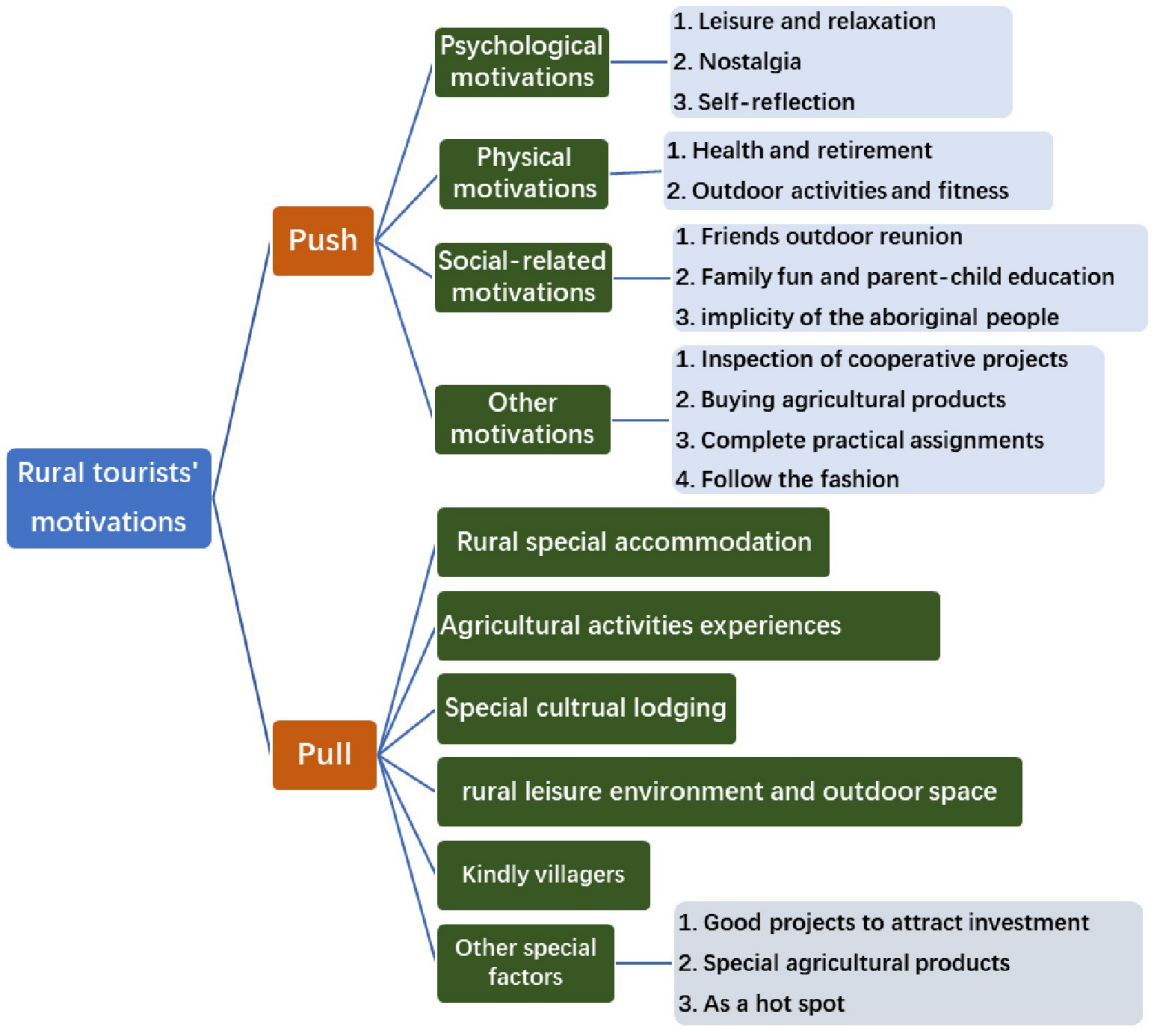
List of push and pull factors of rural tourism motivation.

At present, the motivations of rural tourism mainly involve psychological motivation, physical motivation, social motivation, characteristic experience motivation, and single motivation such as investigating cooperative projects and helping children to finish their homework. In the interview process, some interviewees could not clearly express their clear motivation, but only expressed that they could not go farther because of the epidemic policy and other restrictions, and they especially wanted to go outdoors, so they could only choose to go to the surrounding rural tourism places, that is, there was no special purpose during the special period, just to escape from the closed environment, and wanting to go outdoors to relax also became the rural tourism motivation. Dann’s "push-pull" motivation theory explains the motivation factors of tourists from both internal and external motivation [17, 21], which is a classic theory in the field of motivation research that is easily understood and generally accepted by a large number of scholars, where the push is generated by the internal psychological factors of tourists, and the pull is generated by the attributes of the destination. Combining the "push-pull" motivation theory framework to sort out the motivation of rural tourism in this study, we found that the current "push factors" of rural tourism mainly include the psychological motivation of leisure and relaxation, nostalgia, self-reflection, and fashion pursuit, the social motivation of activities with friends and family, and the social motivation of health and fitness. The "pull factors" are mainly some rural experience motives, including the natural rural environment, the indigenous folk style, rural special food and accommodation, folk customs, activity experience, and the supply of special agricultural products (Fig 2).

## Conclusion, limitations and future research

### Conclusion

Chinese citizens showed a preference for nature, countryside, and cultural descriptions after the epidemic passed; they also preferred short trips after the epidemic passed [48]. Despite the recent finding of a sustained increase in outdoor rural tourism activities during COVID-19, few studies have explored the psychological responses of tourists during the epidemic and their demand for and behavioral decisions about rural outdoor activities. We sought to explore this gap through empirical research. The motivational factors for tourists’ participation in rural tourism identified in this study include not only the tourists’ own "push" factors but also the "pull" factors of rural tourism destinations, which supports Dann’s "push-pull" motivation theory [23, 24].

First, we found that tourists’ motivation to participate in rural tourism activities restricted by COVID-19 threats and government regulations affects tourists’ behaviors in terms of activity choice, companionship preferences, and length of stay. This study impacts the existing literature in three ways. One of the first qualitative studies to conduct an in-depth analysis of tourists’ travel needs during the COVID-19 period, this study found that proximity, outdoor, and safe rural destinations are popular, which combines with people’s need for escapism and their need for safety. Secondly, we outside found the fact that a single motivation is enough to attract tourists to rural tourism destinations, a finding that inspires rural tourism destinations and their operators to create distinctive boutique products to attract demand through precise marketing while increasing awareness. The third significance of this study is academic: it implies that the motives found in this study can enrich the theoretical model of "push-pull" motives, and furthermore, the fact that single motives are widely present in rural tourism activities during the epidemic can be used to develop new theoretical models for future tourism.

### Limitations

Among the rural tourists interviewed, most were from Anhui Province, and very few were from other provinces. Since Chinese people are more introverted or shy, and the research process was conducted in the context of the epidemic, some of the interviewees may have self-importance and social preference bias in the communication process compared to before the epidemic, and were not able to fully express their inner thoughts, and there does not seem to be an effective way to eliminate this bias. Therefore, in this study, both semi-structured interviews and focus group discussion was used in combination, and the technique was used as triangulation.

Currently, China has a "dynamic zero" policy for new infections, during which respondents experience a period of anxiety during home quarantine, a period of release and compensation during the initial period of release, and a period of recovery from normal life. During the data collection period, some of the respondents were in the initial release and compensation period of the release from isolation, and they overemphasized their desire to escape from their usual environment and get some fresh air, choosing the countryside around their residence because they could not go to other provinces and cities. During the researcher’s interview, on the one hand, he or she asked follow-up questions at the right time to get the respondents to say as much as possible, and on the other hand, he or she repeated the key elements described by the respondents in order to obtain affirmative responses from them.

### Future research

In future studies, the knowledge of rural tourist psychological experience research can be further enriched by adding interviews with rural tourists with different demographic characteristics and types of consumption behavior, and adding corresponding interviews to categorize and study the tourism motivations of different categories of rural tourists.

The purpose sampling and snowball sampling techniques used in this study still yielded static cross-sectional data, and respondents expressed their willingness to revisit after their needs were met and they had a good rural tourism experience, but willingness should not necessarily guarantee actual behavior [56]. Later studies can further explore the fit between the subjective responses and behavioral characteristics of rural tourists and the actual behaviors they produce by comparing them over a relatively long study period through more rigorous sample tracking to identify the points of difference and analyze the reasons for them.
